# An insight into real-world practice: What information sources inform the prescribing for children with cancer in Scotland? A focus on emesis, tumour lysis syndrome (TLS), and *Pneumocystis Pneumonia* (PCP) prophylaxis

**DOI:** 10.1177/10781552251392124

**Published:** 2025-10-30

**Authors:** Rahaf Alkhlaifat, Natalie M Weir, Tanja Mueller

**Affiliations:** 1 Strathclyde Institute of Pharmacy and Biomedical Sciences, 3527 University of Strathclyde, Glasgow, UK; 2 59178 Mutah University, Karak, Jordan

**Keywords:** children, information, prescribing, medicines, guidelines

## Abstract

Within clinical practice, information sources such as clinical guidelines ensure practitioners apply evidence-based information, supporting both effectiveness and safety of the prescribed medicines. Paediatric cancer — a leading cause of death in children — is a complex condition, thus is an area where clinical guidelines are important to give guidance on, e.g., recommended doses, drug dispensing, and toxicity monitoring measures. However, exploring clinical guidelines/information sources and their content regarding medicine-pertaining aspects is rarely done despite the fact they directly inform patient care. This comment summarises the main findings of a document analysis study aimed to describe available documents and their content for Scotland-based paediatric prescribers in oncology wards and provides insights into the documents' comprehensiveness and consistency. The analysis covered three clinical indications which are either highly prevalent or troublesome in practice: emesis, tumour lysis syndrome (TLS), and Pneumocystis Pneumonia (PCP) prophylaxis.

Globally, prescribing of medicines is a key intervention within healthcare systems. Medicine prescribing involves two steps: selecting appropriate options for a particular clinical indication; and reaching consensus among relevant stakeholders on a treatment decision.^1^ Making an appropriate prescribing decision — which is fundamental to ensure patient safety^2^ — is complex for children due to different reasons including ethical considerations, children vulnerability,^1^ heterogeneous response to medicines among children,^3^ and the lack of evidence on medicine use in children.^4^ Cancer —as a complex condition—and its traumatising treatment jointly compromise children health, making prescribing more difficult.^1^

The prescribing of medicines within paediatric cancers involves managing distressing side effects and symptoms. Some symptoms like emesis could impair patients’ quality of life and secondarily affect their functional development.^5^ Emesis is developing in a cluster with other ailments like anorexia which might stunt the growth of children, and hence affect children's functional development.^5^ Other conditions like tumour lysis syndrome (TLS), a metabolic emergency, could be life-threatening.^6^ Infections such as *Pneumocystis Pneumonia* (PCP) might be linked to infection-attributable mortality considering the immunosuppressant status of children with cancer.^7^

Prescribers refer to evidence-based information not only to ensure the effective and safe prescribing of medicines but also to discard medicines appropriately. Inappropriate disposal of medicines would lead to environmental pollution.^8^ Information sources and their content are changing continuously, to align with scientific advancements, in the era where all kinds of information are susceptible to change.^9^

## The crucial role of clinical guidelines /information sources: Key drivers of high-quality care

Information sources such as clinical guidelines serve as a link between the meticulously appraised scientific evidence and clinical practice.^10^ Adherence to applicable evidence-informed clinical guidelines should be championed since it prevents unnecessary variation in practice, which has been framed by Edward Demming as “the enemy of quality”^11^ in the provision of care. Further, clinical guidelines improve treatment outcomes in terms of safety and effectiveness by transferring well-tested evidence into action.^10^ From an economic perspective, following clinical guidelines could reduce treatment expenditure.^12^ Other evidence-based sources such as local drug formularies are also designed to promote safe, effective, and cost-effective utilisation of medicines.^13^

## A document analysis: Exploring information sources for prescribers in Scotland

Work is ongoing in Scotland to deliver equitable, optimal, high-quality treatment to children with cancer.^14^ Prescribing is the most implemented intervention within Scottish clinical wards to treat the diagnosed conditions.^15^ The act of prescribing would be supported by “building blocks” such as clinical guidelines and formularies.

**Figure 1.**
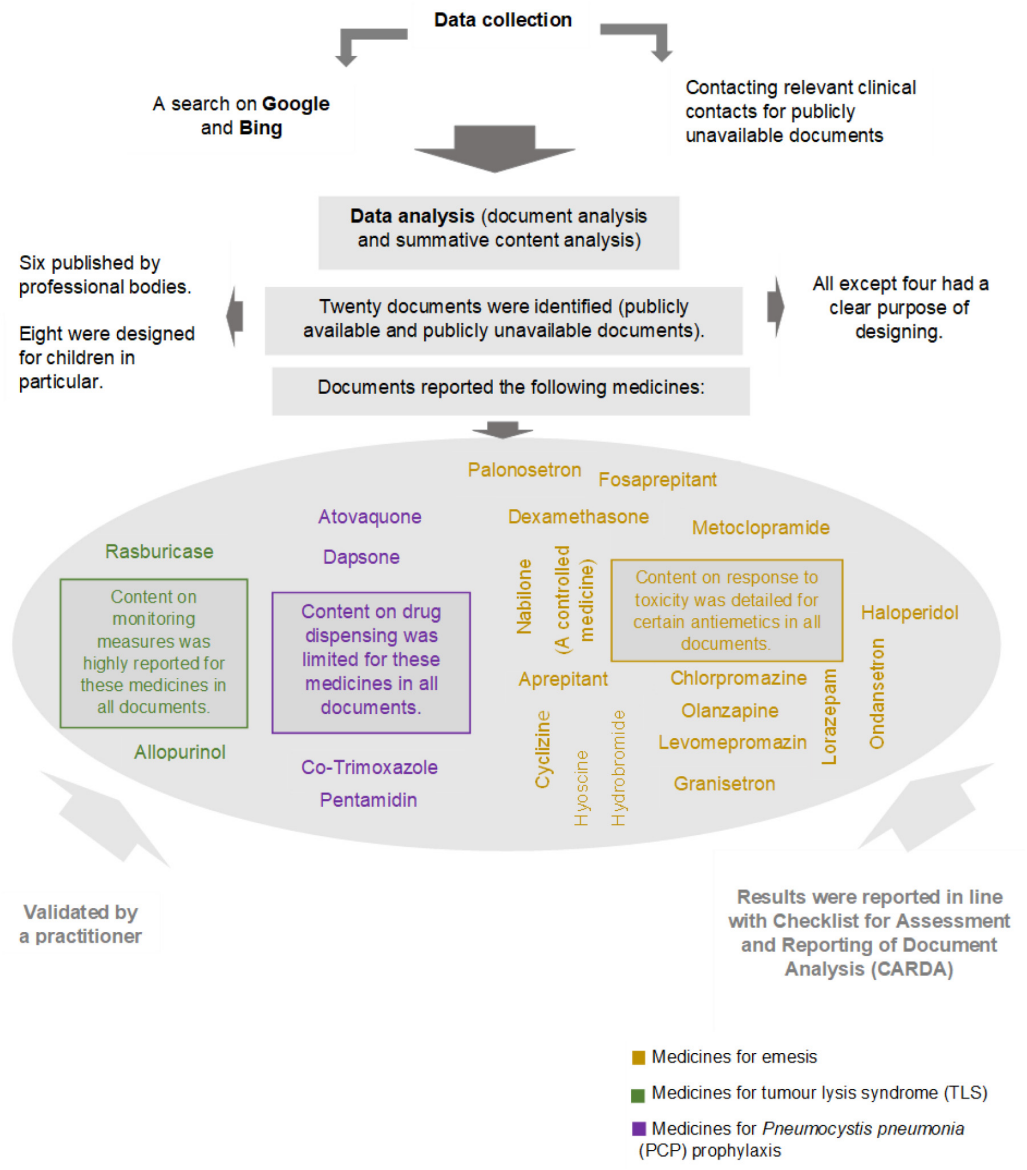
The graphic outline of the document analysis: methodology and identified content.

The current uncertainty about the existing prescribing information sources and their content might make the goal of providing high-quality treatment to children partially achievable. This comment presents a document analysis study (Figure 1) of 20 information sources (documents) and their content that might guide the prescribing of medicines for three clinical indications in paediatric oncology: emesis, TLS, and PCP prophylaxis.

Clinical guidelines and drug formularies were the main types of the documents (n = 13, 65%). Other types of documents such as standard operating procedures (SOPs), evidence-based summaries, and clinical trial protocols were identified along with clinical guidelines and formularies. Additional characteristics of the documents are illustrated in Figure 1.

## The key identified content on the three clinical indications

Regardless of the indication, the content on the recommended doses was the most presented content across national and regional documents (n = 104), which varied in instances. The highest variation between regional and national documents was apparent in the recommended doses for antiemetics. For the other two groups of medicines, variation of the recommended doses was reported for one anti-TLS and one PCP-prophylactic medicine. These variations might be attributed to the heterogeneity in the recommended dosing approaches (i.e. calculating the doses per kg or per body surface area or adapting age bands for dosing). The statements on drug disposal were lacking across all documents for all indications. These statements mostly gave the instructions to follow the local disposal instructions. Information about drug dispensing in all documents generally showed little information on how to dispense low doses of medicines in the presence of high-dose formulations (e.g. rounding the dose to the nearest strength when dispensing).

The guidance on the management of emesis was multidimensional (e.g. considering previous exposure to certain systematic anticancer therapy (SACT) agents and diagnosis with certain malignancies (e.g. lymphoma)). It was evident that recommendations were mostly provided for chemotherapy-induced emesis. Emesis treatment plans for this type were categorised based on the emetogenicity of the planned or given SACT regimens/agents. The number of recommended antiemetics ranged from 3–4 antiemetics for very high emetogenic SACT agents to none for minimal emetogenic SACT agents. Emesis is also triggered by raised intracranial pressure, surrounding environment, administered opiate, and received radiotherapy. Limited content was available on the management of emesis developing from these four stimuli. The lists of recommended antiemetics varied marginally among emesis-guiding documents (Figure 1). No differences were shown in the lists of recommended medicines for TLS and PCP prophylaxis across the relevant supporting documents. For PCP prophylaxis, monotherapy was recommended. The recommendations for the second line option are influenced by the preference of prescribers and patient considerations. The advice on management of TLS emphasised on the need for adequate precautions such as recommending the administration of anti-TLS medicines for mild- and high-risk cases.

## Bridging the gap between what is known and what is unknown and rooms for improvement

Overall, this comment provides additional knowledge about the available evidence-informed documents/their content for prescribers in three clinical areas in Scotland. Several “at hand” evidence-driven information sources including clinical guidelines could support the safe and effective prescribing for children with cancer. Providing additional content on some information-lacking areas such as some types of emesis would quicken the prescribing cascades. Multi-sector efforts should be synergised to provide high-quality and consistent care for children, starting from providing “crystallised” information to prescribers. This would be achieved by creating easy-to-use documents that display the needed information clearly considering prescribers’ top priorities while prescribing.

## References

[1] Bannan DF Aseeri MA AlAzmi A , et al. Prescriber behaviours that could be targeted for change: an analysis of behaviours demonstrated during prescription writing in children. Res Soc Adm Pharm 2021; 17: 1737–1749. 10.1016/j.sapharm.2021.01.007 33514496

[2] Avery AJ Rodgers S Franklin BD , et al. Research into practice: safe prescribing. Br J Gen Pract 2014; 64: 259–261. 24771835 10.3399/bjgp14X679895 PMC4001139

[3] Stephenson T . How children's responses to drugs differ from adults. Br J Clin Pharmacol 2005; 59: 670–673. 15948930 10.1111/j.1365-2125.2005.02445.x PMC1884865

[4] Kimland E Odlind V . Off-label drug use in pediatric patients. Clin Pharmacol Ther 2012; 91: 796–801. 22472984 10.1038/clpt.2012.26

[5] Ruggiero A Rizzo D Catalano M , et al. Acute chemotherapy-induced nausea and vomiting in children with cancer: still waiting for a common consensus on treatment. J Int Med Res 2018; 46: 2149–2156. 29690798 10.1177/0300060518765324 PMC6023075

[6] Cheung WL Hon KL Fung CM , et al. Tumor lysis syndrome in childhood malignancies. Drugs Context 2020; 9: 1–14. 10.7573/dic.2019-8-2 PMC7048108 32158483

[7] Shankar SM Nania JJ . Management of Pneumocystis jiroveci pneumonia in children receiving chemotherapy. Pediatr Drugs 2007; 9: 301–309. 10.2165/00148581-200709050-00003 17927302

[8] Tong AY Peake BM Braund R . Disposal practices for unused medications around the world. Environ Int 2011; 37: 292–298. 20970194 10.1016/j.envint.2010.10.002

[9] Magrabi F Coiera EW Westbrook JI , et al. General practitioners’ use of online evidence during consultations. Int J Med Inf 2005; 74: 1–12. 10.1016/j.ijmedinf.2004.10.003 15626631

[10] Busse R Klazinga N Panteli D , et al. Improving healthcare quality in Europe: characteristics, effectiveness and implementation of different strategies*.* Copenhagen, Denmark: European Observatory on Health Systems and Policies, 2019. 31721544

[11] White CJ . Transitioning from volume to value in cardiovascular care. Cardiovasc Interv 2021; 14: 2738–2743. 10.1016/j.jcin.2021.08.057 34949399

[12] Kosimbei G Hanson K English M . Do clinical guidelines reduce clinician dependent costs? Health Res Policy Syst 2011; 9: 24. 21679458 10.1186/1478-4505-9-24 PMC3128844

[13] McLean D . Medicines formularies: and your point is? Clin Risk 2015; 21: 116–120.

[14] Scottish Government . Collaborative and Compassionate Cancer Care: cancer strategy for children and young people 2021–2026: Scottish Government 2021, https://www.gov.scot/publications/collaborative-compassionate-cancer-care-cancer-strategy-children-young-people-scotland-20212026/pages/4/.

[15] The Scottish Government . Primary care services. 2021.

